# Needlestack: an ultra-sensitive variant caller for multi-sample next generation sequencing data

**DOI:** 10.1093/nargab/lqaa021

**Published:** 2020-04-20

**Authors:** Tiffany M Delhomme, Patrice H Avogbe, Aurélie A G Gabriel, Nicolas Alcala, Noemie Leblay, Catherine Voegele, Maxime Vallée, Priscilia Chopard, Amélie Chabrier, Behnoush Abedi-Ardekani, Valérie Gaborieau, Ivana Holcatova, Vladimir Janout, Lenka Foretová, Sasa Milosavljevic, David Zaridze, Anush Mukeriya, Elisabeth Brambilla, Paul Brennan, Ghislaine Scelo, Lynnette Fernandez-Cuesta, Graham Byrnes, Florence L Calvez-Kelm, James D McKay, Matthieu Foll

**Affiliations:** 1 Genetic Cancer Susceptibility Group, Section of Genetics, International Agency for Research on Cancer (IARC-WHO), 150 cours Albert Thomas, 69008 Lyon, France; 2 Genetic Epidemiology Group, Section of Genetics, International Agency for Research on Cancer (IARC-WHO), 150 cours Albert Thomas, 69008 Lyon, France; 3 Institute of Hygiene and Epidemiology, Charles University, 1st Faculty of Medicine, 116 36 Prague, Czech Republic; 4 Faculty of Health Sciences, Palacky University, 775 15 Olomouc, Czech Republic; 5 Department of Cancer Epidemiology and Genetics, Masaryk Memorial Cancer Institute, 656 53 Brno, Czech Republic; 6 International Organization for Cancer Prevention and Research (IOCPR), 11070 Belgrade, Serbia; 7 Russian N.N. Blokhin Cancer Research Centre, 115478 Moscow, The Russian Federation; 8 Centre Hospitalier Universitaire de Grenoble Département d’Anatomie et Cytologie Pathologiques, CS 10217 38043 Grenoble, France; 9 Section of Environment and Radiation, International Agency for Research on Cancer (IARC-WHO), 150 cours Albert Thomas, 69008 Lyon, France

## Abstract

The emergence of next-generation sequencing (NGS) has revolutionized the way of reaching a genome sequence, with the promise of potentially providing a comprehensive characterization of DNA variations. Nevertheless, detecting somatic mutations is still a difficult problem, in particular when trying to identify low abundance mutations, such as subclonal mutations, tumour-derived alterations in body fluids or somatic mutations from histological normal tissue. The main challenge is to precisely distinguish between sequencing artefacts and true mutations, particularly when the latter are so rare they reach similar abundance levels as artefacts. Here, we present needlestack, a highly sensitive variant caller, which directly learns from the data the level of systematic sequencing errors to accurately call mutations. Needlestack is based on the idea that the sequencing error rate can be dynamically estimated from analysing multiple samples together. We show that the sequencing error rate varies across alterations, illustrating the need to precisely estimate it. We evaluate the performance of needlestack for various types of variations, and we show that needlestack is robust among positions and outperforms existing state-of-the-art method for low abundance mutations. Needlestack, along with its source code is freely available on the GitHub platform: https://github.com/IARCbioinfo/needlestack.

## INTRODUCTION

Massive parallel sequencing, or next-generation sequencing (NGS), has revolutionized the manner in which genetic variation can be explored, due to a large increase in throughput and unprecedented ability to detect low-abundance variations compared to the traditional Sanger sequencing, and at a greatly reduced cost per sequenced base. However, because these new technologies are prone to errors, identifying genetic variants from NGS data remains a considerable challenge (1). This is particularly true in heterogeneous samples, where the variant allelic fractions (VAF, the ratio of the number of sequencing reads carrying the mutant allele to the total read count) deviate away from the expectations of a diploid genome (0, 50 or 100% for the three possible diploid genotypes), until the point where the mutant alleles make up only a small fraction of the sequenced reads, approaching the background error rate. Nevertheless, robustly identifying low VAF sequence variants in such heterogeneous settings can be highly informative, for example providing insights into the clonal evolution of tumours (2), analysing the cell-free DNA in order to identify tumour-derived molecular footprints (3) or evaluating somatic mutations in histologically normal material (4).

The error rate of NGS is known to vary along the genome and even across the different possible base changes at a given DNA position (5,6). NGS errors originate from many of the steps in the sequencing process, stemming from the quality of the template DNA, its subsequent fragmentation, the library preparation, the base calling or the alignment step subsequent to the sequencing of raw reads. Some of these errors have a tendency to reoccur consistently across samples whereas others have a more unpredictable appearance. Because errors in NGS come from multiple sources, it becomes highly difficult to distinguish them from real genetic variation (7). Variant identification methods that consider this highly variable error pattern may improve our ability to robustly detect true sequence variants even when their abundance is low. Most current algorithms use a probabilistic model on VAF applied independently across samples to distinguish between sequencing artifacts and true variations (8), while methods specifically designed to detect low abundance mutation, like shearwaterML (9,10), propose to benefit from the shared knowledge on errors across samples, but are limited by the requirement of a prior threshold on the error rate.

Here, we have explored the approach of using multiple samples analysed concurrently to develop an error model for each potential base change. Sequence variants are identified as outliers relative to this robust error model. This method, called needlestack, allows the identification of sequencing variants in a dynamic manner relative to the variable error pattern found in NGS data, and is particularly appropriate to call variants that are rare in the sequenced material. By combining this method with additional laboratory processing for further error correction (11) and very deep NGS, we are able to robustly identify VAFs well below 1% while maintaining acceptable false discovery rates. We conducted multiple rigorous performance estimations and comparisons with methods for both somatic and germline variant detection. We deployed our pipeline focusing on efficiency and robustness using the domain-specific language (DSL) nextflow (12), and on reproducibility by providing Docker and Singularity images. Source code is versioned and freely available on GitHub (https://github.com/IARCbioinfo/needlestack).

## MATERIALS AND METHODS

### Needlestack overview

Needlestack estimates for each candidate alteration, i.e. each pair of position and base change (the three non-reference nucleotides and each observed insertions and deletions) the systematic sequencing error rate across a series of samples, typically more than twenty to ensure a reasonable estimation of this metric. Then, for each sample, it computes the *P*-value for the observed reads under the null hypothesis of this estimated model of errors, and transforms this *P*-value into a Phred-scale *Q*-value reported as a variant quality score (QVAL) for the candidate mutation. As such it measures the evidence that the observed mutation is not explained by the error model, and should therefore be considered a mutation.

Needlestack takes as input a series of BAM files, and is based on three main piped processes, the generation of the mpileup file containing read counts at the target positions using samtools (13), the reformatting of this file into readable tabulated file and finally the estimation of the error model using our R regression script (see below) coupled with the computations of Q-values (Supplementary Figure S1). Needlestack is highly parallelizable as input positions are analysed independently. As an output, needlestack provides a multi-sample VCF file containing all candidate variants that obtain a QVAL higher than the input threshold in at least one sample, general information about the variant in the INFO field (e.g. error rate estimation, maximum observed QVAL) and individual information in the GENOTYPE field (e.g. QVAL of the sample, coverage of the sample at the position). Other known systematic biases can be filtered out by needlestack in order to reduce the amount of false positive calls: needlestack can filter low quality sequenced bases (BQ filter), reads with low mapping quality (MQ filter) and variants are annotated for strand bias measures in the VCF file. In addition, if needlestack is launched providing pairs of tumour and matched normal samples, it will add a status (i.e. somatic or germline) to each detected mutation (see Supplementary Methods for details).

### The Needlestack algorithm

Let *i = 1…N* be the index of the sample taken from an aligned sequenced panel of size *N*, *j* the genomic position considered and *k* the potential alteration, with $k \in ( {{\rm{A,T,C,G,ins,del}}} )$, *ins* and *del* covering respectively every insertion and deletion observed in the data at position *j*. Let ${\rm{D}}{{\rm{P}}_{{\rm{ij}}}}$ denote the total number of sequenced reads at position *j* for the sample *i*, ${\rm{A}}{{\rm{O}}_{{\rm{ijk}}}}$ the reads count supporting alteration *k* and ${e_{{\rm{jk}}}}$ the corresponding error rate. We model the sequencing error distribution using a negative binomial (NB) regression (without intercept):

$$\begin{equation*}{\rm{A}}{{\rm{O}}_{{\rm{ijk}}}}{\rm{\sim NB}}\left( {{\mu _{{\rm{ijk}}}}{\rm{,}}{{\rm{\sigma }}_{{\rm{jk}}}}} \right) \end{equation*}$$

with ${\sigma _{{\rm{kj}}}}$ the over-dispersion parameter and ${\mu _{{\rm{ijk}}}}\ {\rm{ = }}{{\rm{e}}_{{\rm{jk}}}}\ *{\rm{D}}{{\rm{P}}_{{\rm{ijk}}}}$ corresponding to the expected number of reads supporting alteration *k* across samples with a coverage ${{\rm DP}_{{\rm{ijk}}}}$. A robust negative binomial regression method (14) is employed to ensure that the outliers from this error model, such as true mutations, are not biasing the regression parameters estimates. This model is based on a robust weighted maximum likelihood estimator (MLE) for the over-dispersion parameter ${\sigma _{{\rm{jk}}}}$. We modified the original implementation of this regression to fit the need of our model here with: (i) a linear link function, (ii) a zero intercept, as a null coverage will exhibit a null read count and (iii) an approximation of the bounding functions to allow the MLE to run efficiently for high coverage data (see Supplementary Methods).

For each position *j* and alternative *k*, we perform this robust negative binomial regression to estimate parameters ${e_{{\rm{jk}}}}$ and ${\sigma _{{\rm{kj}}}}$. We then consider a sample *i* as carrying a true mutation *k* at the position *j* when being an outlier from the corresponding error model. We calculate for each sample a *P*-value for being an outlier using the estimated parameters that we further transform into *q*-values using the Benjamini and Hochberg procedure (15) to account for multiple testing and control the false discovery rate.

Importantly, because true mutations are identified as the outliers from the error model fitted using a robust regression, this approach is more suited to detect low-abundance mutations. Common mutations (for example germline SNPs with common allele frequencies) will be observed in the error model and therefore not detected as outliers by needlestack. In practice we found that mutations with a minor allele frequency below 10% can be accurately detected (see below). Additionally, while allowing over-dispersion, our model assumes that the error rate ${e_{{\rm{jk}}}}$ is homogeneous across samples for a given alteration. This means that it should be applied to a homogeneous series of samples (that is prepared using comparable laboratory techniques and sequencing machines etc.). Importantly other types of errors that have less tendency to reoccur uniformly across samples are identified by needlestack as outliers.

### Sequencing data for performance evaluation

One hundred and twenty-five cell-free DNA (cfDNA) samples from healthy donors were used to study the distribution of error rates estimated by needlestack and to estimate its accuracy to detect low VAF using *in-silico* mutations. We also sequenced 46 cfDNA samples from 18 small-cell lung cancer (SCLC) patients and 28 squamous-cell carcinoma (SCC) patients, two cancer types that harbour a high prevalence of *TP53* mutations (respectively 99% (16) and 81% (17)). In order to validate in the tumour the low VAF mutations identified by needlestack in the cfDNA, we also sequenced tumour samples for these patients. Each of the cfDNA samples was sequenced for the *TP53* exonic regions (exons 2–11, which corresponds to 1704 bp with a median coverage of around 10 000×) using the IonTorrent Proton technology, in two technical independent duplicates in order to account for potential errors during library preparation. Details about cfDNA sequencing steps and tumour sequencing method are provided in the Supplementary Material.

Additionally, we performed whole-exome sequencing (WES) from the blood of 62 samples from an independent cohort in order to estimate the performance of needlestack on germline mutations. As a gold standard, we used genotypes derived from Illumina SNP array (Illumina 5M beadarray) that were available for 33 of these 62 samples.

### Comparison with other variant callers

We used BAMsurgeon software (18) to introduce single nucleotide variations (SNVs) at varied VAF in the 125 cfDNA samples in *TP53* in order to benchmark and compare the method through *in-silico* simulations. BAMsurgeon presents the advantage of synthetic benchmarking methods that allow the simulation of mutations for which gold standards do not exist to evaluate the performance (here low VAF, that are in addition challenging to validate), while maintaining the real data background such as the true error profiles. We introduced 1000 SNVs at random positions in the gene in random samples, and we replicated this process in ten batches. As each sample has been sequenced twice, we introduced each *in-silico* mutation in the two technical duplicates of a sample. We took benefit from the variable coverage among samples and genomic positions to study the sensitivity of our method down to VAF = 10^−4^. For each mutation *m*, the VAF was simulated using a log-uniform distribution: ${\rm{VA}}{{\rm{F}}_m}\ {\rm{ = 1}}{{\rm{0}}^{ - u}}$ with $u{\rm{\sim uniform}}( {{\rm{0,4}}} )$. Mutations were only introduced at positions where at least five mutated reads would be observed. This means that a mutation with a VAF = 10^−4^ would be introduced only in positions with a coverage of at least 50 000×. To compare needlestack with a similar variant caller, we ran ShearwaterML (4,10) on the same ten batches (see Supplementary Methods). ShearwaterML is based on a beta-binomial regression and requires an *a-priori* threshold *t* for the error rate. ShearwaterML excludes each sample having a number of alternative bases higher than t*coverage, aiming at removing potential true mutations that act as outliers in the regression to robustly estimate the error rate. To compute the global performance of both methods, the 10 simulation batches were merged, and only mutations detected in both technical duplicates were considered. *In*-*silico* simulations were repeated for 1-bp insertions and deletions (indels) for needlestack. In this case, the total number of *in-silico* mutations was reduced to minimize the potential alignment artefacts created by the introduction of two indels close together. For that, using the same initial data, 100 insertions and 100 deletions were added again in ten simulations batches (total of 20 batches).

To estimate the ability of needlestack to detect rare germline variations, i.e. germline mutations present in a small proportion of the analysed samples, we used the 62 WES from blood samples. Needlestack variant calling was performed using our germline recommendations (see Supplementary Methods). GATK variant calling was performed using HaplotypeCaller best practice workflow (19) (see Supplementary Methods). From the 3 446 898 bead array non-reference genotypes (0/1 or 1/1) distributed over 113 232 positions in the 33 individuals, we selected 20 439 genotypes with a sufficient coverage (see Supplementary Methods). In a second part, to account for possible bias in the array, variant calls from both needlestack and GATK were compared independently of the array data, on a total of 44 314 972 exonic positions. To compare only rare germline variants, we removed common variants from each calling set (bead array, GATK calling and needlestack calling, see Supplementary Methods). Note that while the main goal of needlestack is to accurately detect low VAF mutations, the comparison with germline calling where VAF = 50% or 100% aims at evaluating the performance of needlestack in a very well-studied problem for which a gold-standard is available.

### Error rate estimation

To estimate the error rate variability across positions, we computed with needlestack the sequencing error rates from two data sets of the *TP53* gene sequenced with two different technologies (on the 62 blood samples and on the 125 cfDNA samples). Error rates were estimated at each position of the gene and for each substitution, totalling 1704*3 = 5112 values. We were then interested in estimating the contribution of each possible nucleotide change on the error rate. We therefore computed, for each error-rate range *e* in [{10^−5^,10^−4^};{10^−4^,10^−3^};{10^−3^,10^−2^};{10^−2^,10^−1^}] and for each possible base change *b* in [G>T, C>A, …, A>C, T>G]:

$$\begin{equation*}\ pro{b_{e,b}} = \frac{{{\rm{\# }}E{R_{e,b}}}}{{{\rm{\# }}E{R_e}}}\ \end{equation*}$$

with ${\rm{\# }}E{R_{e,b}}$ being the number of estimated error rates in the class *e* observed for a base change *b*, and ${\rm{\# }}E{R_e}$ being the total number of estimated error rates in the class *e*.

In the case of the Ion Torrent sequencing, we observed a sufficiently high number of single nucleotide variations (SNVs) (*n* = 5112) to also compute the distribution of error rate depending on the 96 possible SNVs taking into account the preceding and following bases to evaluate the effect of the sequence context. Similarly, the high number of insertions (*n* = 7662) and deletions (*n* = 1724) detected allowed us to also compute the distribution of estimated error rates (i) as a function of the length of the inserted/deleted sequences; and (ii) as a function of the length of homopolymer regions for the insertion/deletion of one base pair.

### cfDNA and matched tumour analysis for validation

Observed deleterious mutations in the *TP53* gene of a cfDNA lung cancer patient are generally expected to be derived from their tumour (but see 20). Therefore we used the tumour samples as a proxy for validation of the identified cfDNA mutations. To limit our false discovery rate, samples that harboured a high number of raw mutations (>100) in at least one of the two technical replicates were excluded. This removed four SCC and seven SCLC from the 46 matched samples. We considered only cfDNA mutations that passed post-calling filters, i.e. a RVSB (Relative Variant Strand Bias) (20) lower than 0.85, no high-VAF variant (i.e. a VAF 10 times higher than the candidate mutation) within 5 bp upstream or downstream, and a VAF higher than 10% if the mutation is found in a low confidence base change (i.e. where technical duplicates don’t cluster together; see Supplementary Methods). We independently performed the needlestack variant calling on the cfDNA samples and the matched tumour samples.

## RESULTS

### Sequencing error rates depend on the alteration type

**Figure 1.**
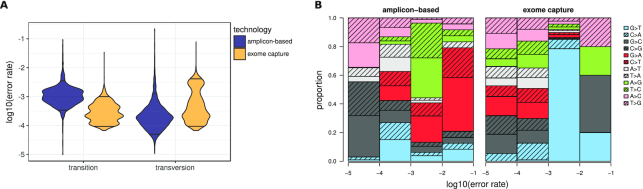
Sequencing error rates estimated by needlestack across the *TP53* gene. (**A**) Distribution of sequencing error rates in log_10_ scale across the 1704 positions accounting for a total of 5112 values. Results are stratified by type of base change: transition or transversion (*x*-axis) and by sequencing technology (IonTorrent Proton amplicon-based data in violet and Illumina exome capture data in yellow). Horizontal black lines correspond to the 5% quantiles of each of the sequencing error rate distribution. (**B**) Contribution of each of the 12 possible base changes on the estimated error rate. Error rates are stratified by ranges ([10^–5^,10^–4^];[10^–4^,10^–3^];[10^–3^,10^–2^];[10^–2^,10^–1^], *x*-axis). Base change contributions are coloured according to DNA strand equivalences (e.g. G to T and C to A are both coloured in blue).

Globally, 95% of the error rates across alterations were estimated as lower than 10^−2.5^ in both sequencing technologies (Figure 1A). Nevertheless, the error rates varied importantly across the target sequences and alterations. For the amplicon-based Ion Torrent sequencing, transitions had 5-fold higher error rates than transversions (Figure 1A), on average, although not clearly influenced by the sequence context when considering the flanking 3′ and 5′ bases (Supplementary Figure S2). For exome-capture sequencing, a bulk in the distribution of transversion-like errors is observed at an error rate in the order of 10^−2.5^ (Figure 1A). When looking at the proportion of different nucleotide substitutions across multiple ranges of sequencing error rates (Figure 1B), we observed that in this range (10^−2^ – 10^−3^) the majority of substitutions correspond to G>T transversions, previously reported and suggested to be related to DNA sonication (21).

As previously reported, we observed a large number of indels (9389) in the Ion Torrent sequencing data (22). We found that the error rate is dependent of their length: long indel (with a size >3 bp) error rates are around 100-fold lower than 1 bp indel error rate (Supplementary Figure S3A). As previously reported (22), the error rate also increases with the length of homopolymer region, reaching 1% for repetitions of 4 nucleotides (Supplementary Figure S3B).

### Variant detection limit depends on the error rate

**Figure 2.**
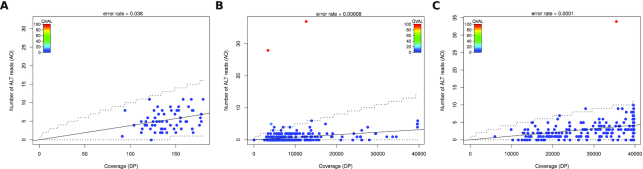
Needlestack regression plot for three independent genomic alterations. Each dot corresponds to a sequencing library of a sample and the dots are coloured according to the *Q*-values attributed by needlestack. Red dots are libraries identified as carrying the mutation by needlestack (their *Q*-values are higher than 50). Dotted lines correspond to 99% confidence interval around the estimated error rate. (**A**) Example of a G to T transversion from exome-hydrid capture Illumina sequencing where the sequencing error rate is estimated as 3.8 × 10^–2^ and no variant is detected. (**B**) Example of a validated mutation (i.e. found in the two technical replicates of the same sample) with a VAF around 10^–3^ with a corresponding sequencing error rate estimated around 10^–4^. (**C**) Example of a non-validated mutation with a VAF at 10^–4^ in the positive library.

Importantly, errors identified in the previous section are classified as such by needlestack, and not as potential variants, even when the error rate is high, as opposed to traditional variant callers which consider samples individually and that rely mostly on the VAF (21). Figure 2A illustrates a position at which needlestack identifies a high error rate (${e_{{\rm{jk}}}} = \ 3.8$) without reporting any variant, even though alternate reads are observed in individuals VAF’s up to ∼9%. Figure 2B illustrates a position with a very different estimated error rate (${e_{{\rm{jk}}}} = {\rm{1}}{{\rm{0}}^{ - 4}}$) where a putative low VAF variant is identified. It is also noteworthy that the variant identified in Figure 2B has a VAF 10 times lower (${\rm{1}}{{\rm{0}}^{ - 3}}$) than the error rate estimated in Figure 2A, indicating that the sensitivity to detect a variant is considerably improved at the site with the lower error rate, highlighting the need to quantify the error rate distributions for each candidate mutation independently.

### Technical replicates reduce low VAF false calls

**Figure 3.**
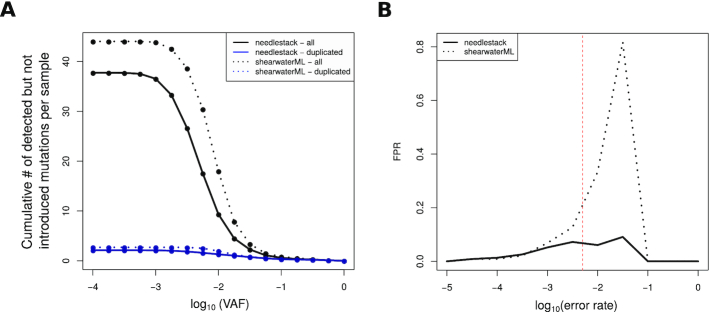
Needlestack and shearwaterML variant calling false discovery overview from *in-silico* simultations with BAMsurgeon on 125 duplicated samples of circulating cell-free DNA from healthy individuals. (**A**) Cumulative number of detected mutations that were not introduced by BAMsurgeon as a function of the VAF (in log_10_ scale) of the mutations, for both methods (needlestack in solid lines and shearwaterML in dotted lines). This number is computed as the average per library when considering all mutations (black lines) and as the average per sample when considering validated mutations (i.e. found in the two technical replicates of the same sample blue lines). (**B**) False positive rate (per alteration) for both needlestack and shearwaterML, depending on the estimated error rate at the position (in log_10_ scale). The red line corresponds to the error rate threshold *t* used for shearwaterML (0.005). ShearwaterML uses this threshold to remove *a-priori* true variants, i.e. samples with a VAF>*t*, to then estimate the error rate.

We noted that the majority of variants detected by needlestack in the cfDNA of healthy patients harbour a particularly low VAF, typically under 0.5% (Figure 3A, black solid line). Importantly, the majority of these variants are not present in a second library preparation (a technical duplicate) of the same sample (Figure 3A, blue lines). Such variants illustrate an additional type of errors found in NGS data that do not consistently re-occur in the samples and that are not validated when sequencing a technical replicate of the sample, for example those introduced by polymerase chain reaction (PCR) amplification errors. These non-systematic artefacts are not expected to be captured by our error model and should be detected by needlestack as outliers (see Figure 2C for such an example). Importantly, we showed that this high number of calls not validated in a technical replicate of the sample is not dependent on our method (Figure 3A, blue lines). Subsequently, here, for the evaluation of needlestack's ability to detect efficiently low VAF mutations, we added the condition that variants are also detected in the technical duplicates to account for this type of error (Figure 3A, blue line).

### Performance evaluation using *in-silico* simulation of somatic mutations

**Figure 4.**
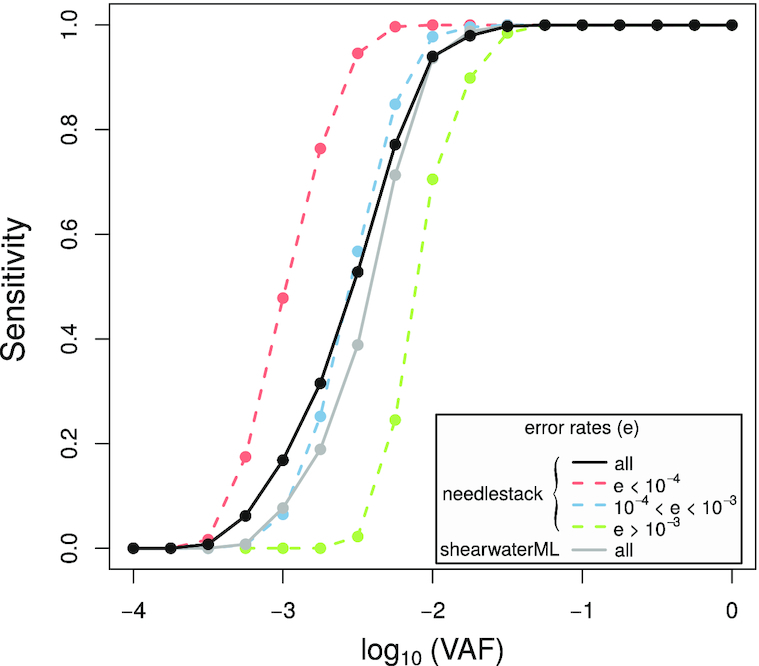
Performance of needlestack for somatic mutation calling using simulated data. The sensitivity of needlestack is shown for multiple values of VAF (in log_10_ scale, *x*-axis) of *in-silico* simulated mutations. A total of 10 × 1000 SNVs were introduced using the BAMsurgeon software, on a set of 125 samples sequenced at the *TP53* gene with the IonTorrent Proton technology. Needlestack sensitivity was computed independently for different error rate ranges (*e*, red, blue and green lines). Black line corresponds to the global sensitivity for all the mutations independently of the sequencing error rate. Global sensitivity of shearwaterML for the same data is shown in grey.

From the 10 000 mutations introduced by BAMsurgeon, needlestack detected 5% of mutations with a VAF < 0.1%, 51.4% of mutations with a VAF between 0.1 and 1%, 99% of mutations with a VAF between 1% and 10% and 100% of mutations with a VAF higher than 10%. As expected, the sensitivity of needlestack is highly dependent on the sequencing error rate. Indeed, needlestack does not call a mutation if the sequencing error rate for that alteration is greater than or in the same range as the VAF of the candidate mutation (Figure 4). As an example, needlestack detected 0, 6.5 and 47.8% of SNVs with a VAF of 0.1% at positions where the sequencing error rate was higher than 0.1%, between 0.1 and 0.01%, and lower than 0.01%, respectively. When comparing needlestack and shearwaterML, we found that globally needlestack sensitivity was higher than that of ShearwaterML, and, for example, ShearwaterML detected 7.7% of all inserted mutations with a VAF at 10^−3^ whereas needlestack detected 16.8% of these mutations. Given *t* the shearwaterML *a-priori* threshold on the sequencing error rate (Figure 3B, red line) and *e* the observed sequencing error rate, we showed that the false positive rate of shearwater is markedly increased when *t* > *e*, whereas needlestack's false positive rate is stable across the whole range of error rates (Figure 3B).

These results show that the key parameters driving the ability of needlestack to detect a variant are the sequencing error rate, the depth of sequencing (DP) and the VAF of the variant. In order to better understand how these parameters influence the sensitivity of needlestack in other possible sequencing scenarios, we have trained a machine learning model on the set of the 10 000 introduced point mutations to predict this sensitivity in the entire space of possible mutations (see Supplementary Methods). The model was trained with only two key features: the number of mutated sequencing reads (AO = DP*VAF) and the ratio between the VAF of the mutation and the sequencing error rate. Interestingly, the ability of this simple model to predict the sensitivity of needlestack was extremely high (area under the curve of the precision-recall curve of 0.998, Supplementary Figure S4A). We therefore applied it on the whole space of parameters to evaluate the sensitivity of needlestack. We observed two main raisons for a lack of sensitivity to detect a mutation with a given VAF: (i) an insufficient coverage for this VAF, with a general guideline is that the number of mutated sequencing reads should be larger or equal than five (log_10_(DP*VAF) > 0.7 on Supplementary Figure S4B), and (ii) an error rate too high for this VAF, with a general guideline is that the VAF should be larger than three times the sequencing error rate (log_10_(VAF/ERR) > 0.5 on Supplementary Figure S4B).

### Specificity of needlestack using simulations of NGS reads

The specificity of needlestack for different scenarios was assessed using simulations of NGS reads that include a realistic sequencing error model and without adding any variants (23) (see Supplementary Methods). We explored four alternative sequencing scenarios based on different average sequencing depths: (i) 30× mimicking a traditional WGS case, (ii) 100× mimicking a traditional WES case, (iii) 1000× mimicking a deep targeted sequencing case and (iv) 10 000× mimicking an ultra-deep targeted sequencing case. We found that the number of false positives by mega-base (FP/Mb) of needlestack decreases with the increase of the QVAL threshold, reaching a virtually null value for QVAL> 60 (Supplementary Figure S5). With QVAL = 50 (the default value for somatic variant calling in needlestack), the specificity was estimated at 0.0 FP/Mb for the WGS setting, 1.67 FP/Mb for the WES, 0.24 FP/Mb for the deep targeted sequencing and 0.48 FP/Mb for the ultra-deep sequencing. The median VAF of the false positive variants decreases with the increase of the average coverage, and was estimated at 4.7% for the WES scenario, 1.2% for the deep targeted sequencing scenario and 0.06% for the ultra-deep sequencing scenario.

### Detection of tumour-derived mutations from cell-free DNA

Next, we tested needlestack's ability to detect very low VAF mutations in a biologically relevant setting, i.e. by validating in the tumour the mutations found in the cfDNA sample of the same patient. For this we screened cfDNA extracted from plasma samples from 35 lung cancer patients where the matched tumour sample was analysed concurrently, and considered the concordance between the identified variants. A total of 22 *TP53* mutations from 18 samples (9 SCLC and 9 SCC) were identified in the cfDNA. A total of 16/22 (70%) mutations were called in the tumour of the same patient. All the 12/22 cfDNA mutations considered as deleterious (i.e. indels, non-synonymous SNVs with a REVEL score higher than 0.5, stopgain or stoploss variants) (24) were present in the tumour. cfDNA and tumour VAF were found to be moderately correlated, which is concordant with previously reported results (25) (Pearson correlation coefficient ρ equals to 0.59, Supplementary Figure S6A). Details of the 22 cfDNA mutations and corresponding observations in the tumour matched samples are provided in Supplementary Table S1. The needlestack plots of a low VAF cfDNA mutation validated in the tumour are shown in Supplementary Figure S6B.

### Application to germline variant calling

**Figure 5.**
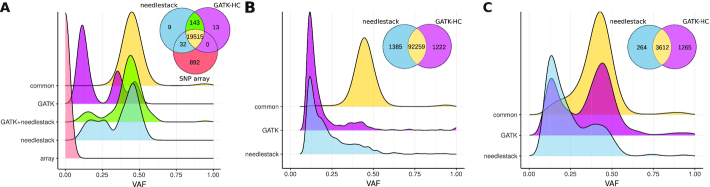
Germline variant calling comparison between needlestack and GATK-HC across 62 samples. Both distributions of the VAF and Venn diagrams showing the concordance of called mutations are shown. VAF distributions are coloured according to the Venn diagram. (**A**) Comparison between both methods and an Illumina bead array containing gold standard genotypes available for a total of 33 samples. (**B** and **C**) Comparison between needlestack and GATK-HC called mutations without any reference gold standard for both SNVs (B) and indels (C).

For rare germline variants from 33 whole exomes, needlestack has a sensitivity of 95.64% to detect non-reference genotypes when using bead array data as a gold standard, which is quite similar to the GATK-HC Haplotype Caller results (95.48%). GATK-HC and needlestack variants concordant with the bead array (19 515 of the 20 439 variants) had VAF distributed around 50 and 100%, as expected for germline variants (Figure 5A). Most of the few calls that were not validated in the array were also centred around 50% and found by both variant callers, implying that they certainly contain additional heterozygotes that the SNP array failed to detect. Finally, the majority of variants not identified with NGS had no sequencing reads supporting the alternative allele detected by the array (841/892 variants), suggesting that these variants are potentially false positive results from the SNP array (Figure 5A).

Because SNP arrays are biased toward sites amenable to the design of Illumina BeadArrays (26), we also undertook needlestack and GATK germline genotyping of SNVs and indels calls across 62 exomes. Respectively, 97.3 and 70.3% of the SNV and indel calls were concordant (Figure 5B and C) with VAFs around 50%, whereas the genotypes identified uniquely by one of the two methods tended to have low VAF. For indel calling, 46% of calls unique to needlestack and 34% of calls unique to GATK are more than 10 bp long, compared to only 12% of common calls. This suggests that discrepancies among the methods can be partially explained by longer indels that are difficult to align and call. For 66% of uniquely called indels by GATK-HC, no alternate reads were present in the BAM file used by needlestack, suggesting divergences in the assembly steps (haplotyper Caller versus ABRA). Interestingly, for 52% of the SNVs detected by GATK-HC and not by needlestack, needlestack estimated an error rate higher than 1%, pointing to possible false positives in the GATK calls (Supplementary Figure S7).

## DISCUSSION

The needlestack method is based on the notion that, as error rates strongly vary along the genome, their dynamic estimation from multiple samples, for each potential base change at a given DNA position, may assist in accurately identifying sequence variants. Here, we have demonstrated that, even within a single gene (*TP53*), and even if the sequencing error rate is generally low, it varies importantly across positions and base changes (Figure 1). Needlestack implements a robust negative binomial regression for this purpose, and the ability of the method to identify variants will be dependent upon the error rate at that particular site and for that base change. By identifying sequence variants as outliers relative to the error model, needlestack maximizes the sensitivity to detect variants in a dynamic manner relative to the error rate in that particular setting. As such, low allelic fraction variants are identified from sites with low errors rates, whereas in settings where error rates are high, needlestack maintains reasonable false discovery rates (Figures 3 and 4).

We have benchmarked our method using both simulated and real data from different sequencing platforms. First, we have tested our method on low VAF mutations using BAMsurgeon to generate *in-silico* mutations and have compared our findings to variants identified by a similar rare variant orientated algorithm shearwaterML (9,10). We have shown that our method outperforms shearwaterML for the detection of mutations with VAF lower than 10^−2^ and that the performance of shearwaterML highly depends on the difference between the error rate *e* and the error rate *a-priori* threshold *t* (see ‘Materials and Methods’ section for details). Contrary to shearwaterML, needlestack's specificity is not dependent on the sequencing error rate. In addition, needlestack also considers small indel mutations (smaller than the length of a short-read sequencing alignment). For this type of variant, the sensitivity of needlestack is slightly reduced compared to SNVs (Supplementary Figure S8A and B). This is potentially due to the increased complexity of the assembly step around indels compared to SNVs. Moreover, needlestack detects a high number of indels replicated in the two technical duplicates that were not *in-silico* introduced (around eight by samples in average), whereas *TP53* is not expected to harbour many indels in healthy patients. These mutations can be moderated using a filter on the strand bias, as previously reported (Supplementary Figure S8C and D) (27). In order to estimate the sensitivity of needlestack for multiple types of target mutations (defined by their VAF) and multiple types of sequencing experiments (defined by the coverage and the error rate), our machine learning model identified a simple rule of thumb to predict the ability of needlestack to detect a mutation (VAF*DP > 5 and VAF > 3*ERR). As the sequencing error rate depends highly on the sequencing technique used and are generally well characterized, these guideline will help users designing their experiments, in particular choosing the sequencing depth for the range of VAF they are looking for.

The true specificity of needlestack cannot be achieved with BAMsurgeon simulations, due to a probably very low presence of true mutations in the cfDNA of healthy patients that is difficult to determine *a priori* (20). In order to estimate the specificity of needlestack in different sequencing scenarios, we have generated simulated data that do not contain any variant for three common sequencing scenarios. We have shown that the false positive rate is directly impacted by the QVAL threshold, and this will help guiding users in their choice. We have also estimated the validation rate in the tumour of deleterious cfDNA mutations identified by needlestack in 35 lung patient cfDNA samples. All of these 12 mutations were validated in the tumour. We detected additional mutations in the tumours that were not detected in the cfDNA of the corresponding patients (27 mutations in total). However, this can’t be used to estimate the sensitivity of needlestack, as several biological factors have been shown to influence the release of tumour DNA in the circulation (organ, tumour stage, necrosis, physical activity etc.; see (28) for a review). Without knowing a priori if some tumour DNA was actually present in the circulating DNA we sequenced, it is impossible to disentangle the ‘biological sensitivity’ of the cfDNA to accurately represent the tumour, from the ‘technical sensitivity’ of our variant calling procedure.

Finally, we have benchmarked needlestack on germline mutations using SNP array data to validate the mutations detected in WES of 33 individuals, and showed an excellent concordance when results are compared with both an SNP array as a gold standard set and calls from GATK HaplotypeCaller. This illustrates that needlestack, even if based on a totally different approach to detect variants, can reach similar performance to state-of-the-art germline variant callers. More importantly, we have shown that needlestack can correct false positive variants called with GATK that correspond to recurrent errors across samples (Supplementary Figure S7). Practically, users can also run needlestack directly using a multi-sample VCF produced by GATK as an input in order to filter out these errors.

Whilst we have performed some extensive evaluations of needlestack, it is worth noting that they only provide indirect measures of performance based on *in*-*silico* mutations, NGS reads simulation, comparison to germline calling with high VAF or biological validation in tumour tissue. Ideally one would need a series of >50 samples, with very deep (10 000×) WES/WGS data, with a list of very low VAF validated variants, and a list of genomic position at which the absence of variants has been biologically confirmed. This would provide a direct measure of sensitivity and specificity, but as of today, biologically validating/invalidating very low VAF variants is both technically challenging and very expensive, as well as performing very deep sequencing in a large series of patient at high coverage. There are ongoing efforts to generate such high-quality gold-standards to evaluate the performance of aligners and variant callers, but as of today they mostly focus on germline (high VAF) variants (29).

The needlestack method nevertheless has several limitations. Even though needlestack is extremely sensitive, it is suited to detect rare mutations rather than common germline polymorphisms or hotspot mutations, which both re-occur at the exact same genomic location across multiple samples. Indeed, we observed that our method is not able to detect a mutation present in more than 20% of the analysed samples. Having a high number of mutated samples breaks the assumption of the robust regression model we use, leading to include mutated samples in the error model, which in tun prevents the detection of these mutations. Adding an *a-priori* threshold for the error rate (extra_robust mode—see Supplementary Methods) can partially offset this limitation, but is only applicable to particular situations for the reasons explained above. More importantly, the inherent logic of the needlestack approach corrects for errors that have a tendency to reoccur, as such errors that are rarer are identified as outliers in the regression. For the same reason needlestack should be applied to a homogeneous series of samples, because batch effect could be a source of false positives. For large studies in which heterogeneity cannot be avoided, we recommend to group and analyse samples in homogenous series separately. Following this, needlestack does not correct for sample-specific artifacts such as (i) (sample specific) stochastic alignment errors and we recommend to use it in conjunction with an assembly based re-alignment method (30); (ii) polymerase errors introduced in PCR amplification step; (iii) complex errors leading to features like strand bias. Such errors remain a feature in NGS data (Figures 2C and 3A), thus additional error correction (31,32) and/or validation techniques are needed. This can be achieved with hard filtering on the output statistics such as the VAF or the strand bias, but also with machine-learning-based approaches applied to multiple variant summary statistics when validated data are available to inform the model. A more elegant solution would be to directly include some of these bias in the needlestack model rather than performing post hoc filtering, but this will require a better modelling of these errors. Here we have controlled for these errors by undertaking technical duplicate of each sample and conditioning on the requirement that the variant must be present in each preparation.

Our pipeline is implemented using nextflow (12), to facilitate its scientific reproducibility but also efficient parallel computations. Needlestack is also provided with Docker (33) and Singularity (34) containers to avoid installation of dependencies and produce perfectly reproducible results. Needlestack is a user-friendly pipeline that can be run in one command line. In addition, needlestack implements a power calculation to estimate if the coverage is sufficient to call a mutation when applied on tumours with matched normal samples to determine the germline or somatic status (see Supplementary Methods for details). Using this power analysis, it can predict the germline or somatic status of a mutation. This also allows needlestack to flag mutations with an ‘unknown’ status (when the coverage is too low) to accurately control the false discovery rate. Source code is available on GitHub and is versioned using a stable git branching model. Importantly, this approach is relatively computationally efficient and parallelizable. This allows error models to be built even across large target stretches of DNA, enabling applications at the exome level, genome levels or to most forms of sequencing data. As an example, needlestack takes around 20 h to analyse 100 WES when launched on 100 CPUs.

In summary, needlestack uses a robust model of sequencing errors to accurately identify DNA mutations potentially in very low abundance. The model takes the advantage of batch sequencing of multiple samples to precisely estimate the error rate for each candidate alteration. Needlestack can be applicable to various types of studies such as cfDNA, histological normal tissue investigation or high-precision tumour subclonality estimation by providing a high sensitivity for low-allelic fraction mutations.

## DATA AVAILABILITY

Needlestack is an open source software and is available in the GitHub repository (https://github.com/IARCbioinfo/needlestack). The exome sequencing data presented in the current publication have been deposited in and are available from the dbGaP database under dbGaP accession phs001971.v1.p1. Targeted-sequencing data have been deposited in the European Genome-phenome Archive database, which is hosted at the EBI and the CRG, under accession numbers EGAS00001003984 (SCLC ctDNA), EGAS00001003985 (SCLC tumours), EGAS00001003987 (SCC ctDNA), EGAS00001003988 (SCC tumors) and EGAS00001003989 (healthy donors cfDNA).

## Supplementary Material

lqaa021_Supplemental_Files
