# Supplementary material for: Needlestack: an ultra-sensitive variant caller for multi-sample next generation sequencing data
Source: NAR Genom Bioinform. 2020 Apr 20;2(2):lqaa021. doi: 10.1093/nargab/lqaa021 (PMC7182099; doi:10.1093/nargab/lqaa021)
Supplement: lqaa021_Supplemental_Files [file lqaa021_supplemental_files.zip › NAR_supp_material_FORMATTED_merged_no_track_changes.pdf]

SUPPLEMENTARY MATERIAL

SUPPLEMENTARY METHODS

Robust Negative Binomial regression

The original method (1) was established on falls data where the predictor variable took values from 0 to a couple of hundreds. Here we need to take into account cases where we sequenced deeply and therefore the predictor variable DP can be up to hundreds of thousands. The first model uses integrals of bounding functions for the maximum likelihood estimation (MLE) of e_{jk} to keep robustness, which can take a very long time for high coverage data. To save computing time, we approximate the calculation of integrals required for the MLE of e_{jk}. Instead of computing the sum of all values corresponding to the integral, we interpolate the points using the *spline* function in R, and compute the sum of a set of sampling points with a reasonable size (default is 100).

The initial estimation of e_{jk} for the MLE algorithm is based on a Poisson model, and because of a lack of robustness the following MLE of σ_{jk} can take a lot of time. We thus define our initiation of e_{jk} as the mean of observed e_{ijk} after passing the Tukey's outlier filter, *i.e* an observed e_{ijk} is taken into account for the mean computation if and only if it verifies $e_{\mathrm{ijk}} \leq Q_3 + 1.5 * \mathrm{IQR}$, with $\mathrm{Qi{=}i^{th}quartile}$ and $\mathrm{IQR{=}Q_3} - Q_1$.

Implementation

Needlestack is implemented as one major process, which can be executed in parallel for multiple input chunks, each corresponding to a set of genomic positions. This process is defined as a chain of piped commands: firstly, it runs samtools mpileup utility to compute, for each of the input BAM files, the list of read nucleotides overlapping the input positions. Then, it translates the samtools output into an easier to process format through a custom C++ tool (mpileup2readcount). Finally, needlestack uses its own R script to run the variant calling independently at each position and for each observed alternative base change, and produces a resulting VCF file that will be merged with other created VCF if run in parallel mode. See Supplementary Figure 5 for details on the pipeline. Needlestack is written in the nextflow (2) domain-specific language, allowing high scalability and reproducibility, but also efficient parallel execution. Needlestack source code is freely available on Github (3), and a Docker container image is hosted on DockerHub (4). This docker image is based on conda and Bioconda (5), a sustainable and comprehensive collection of bioinformatics software that help to easily install workflow dependencies.

Tumour-Normal pairs method

We have implemented, in addition to our basic model, a method to classify any observed variant as somatic or germline when needlestack is launched in tumour-normal pairs mode (Supplementary Table S2). For this, for each variant detected only in either the normal or tumor sample, needlestack estimates the power to detect it in the other sample. Indeed, not observing a variant in the other

sample could be due to the lack of power to detect it, in particular when the depth of coverage is not sufficient. Our power metric is based on the expected Q-value of the variant if truly present, which depends on the observed coverage of the sample at the site, the expected allelic fraction and the error model estimated by Needlestack. If this Q-value is below the user-chosen threshold, we report a lack of power. For a particular individual sequenced for a tumour-normal pair, needlestack classifies its variants as follows: if a variant is observed in the tumour sample, it is classified as "somatic" if not observed in the normal although the power was sufficient, and as "unknown" in a case of a lack of power in the normal sample. If a variant is observed in the normal sample, it will be labeled as "germline", and sub-labeled as "confirmed" if also found in the tumour, "unconfirmed" if not found in the tumour whereas power was satisfactory, and "unconfirmable" if there was not enough power in the tumour to detect it if present.

To obtain the expected Q-value of a variant, the first step is the computation of the minimum expected alternative read count (6) in the other sample (tumor or normal). The expected AO of a (germline) variant in the normal sample is computed as follows:

$$\mathrm{AO_{normal}}=\mathrm{C_{99}}[\mathrm{NB}(\mu,\sigma)]$$

with C_{99} the 99th centile, *NB*=negative binomial distribution, μ=0.5 x coverage at the position, and σ=dispersion parameter (by default=0.1).

The expected AO of a variant in the tumor sample is computed as follows:

$$\mathrm{AO_{tumor}}=\mathrm{c_{99}}[B(\mathrm{n,p})]$$

with C_{99} the 99th centile, *B*=binomial distribution, *n*=coverage at the position, and *p*=minimum variant allelic fraction expected (by default=0.01).

Then, given this expected AO, we compute the expected p-value that corresponds to the probability to belong to the error model that needlestack has estimated. Finally, we transform this p-value into a Phred-scale Q-value to obtain the expected Q-value of the variant.

cfDNA and tumour sequencing for *in-silico* simulations and tumour validation

CfDNA was extracted from 0.8-1.3 mL of plasma using the QIAamp DNA Circulating Nucleic Acid kit (Qiagen) following manufacturer's instructions. CfDNA was eluted into 100 µL of elution buffer and quantified with the Qubit DNA high-sensitivity assay kit (Invitrogen Corporation). Twenty-one amplicons of 150 bp in size were designed (Eurofins Genomics Ebersberg, Germany) to cover exons 2 to 11 of *TP53*. The GeneRead DNAseq Panel PCR Kit V2 (Qiagen) was used for target enrichment. A validated in-house protocol was used to set up multiplex PCRs in 10 µL reaction volume, containing 5 ng cfDNA, 60 nM of primer pool and 0.73 µL of HotStarTaq enzyme. The experiments were carried out in two physically isolated laboratory spaces: one for sample preparation and another one for post-amplification steps. Amplification was carried out in a 96-well format plates DNA engine Tetrad 2 Peltier Thermal Cycler (BIORAD) as follows: 15 min at 95ºC and 30 cycles of 15 seconds at 95ºC and 2 min at 60ºC and 10 min at 72ºC. Two technical duplicates were undertaken for each cfDNA sample including amplification, library preparation, and sequencing. Each technical duplicate pair was assessed on two separate plates to limit the possibility of a contamination.

For the tumour sequencing, eighty nanograms of each DNA sample was used as template to set up four separate PCR reactions (20ng/pool) using the Qiagen GeneRead DNAseq Panel PCR Kit V1 and primer mix (Qiagen), following manufacturer's instructions. The amplified PCR products were then pooled, purified with the Serapure magnetic beads and subjected to library preparation including adapter ligation, purification, and amplification using the NEBNext Fast DNA Library Preparation Kit (New England Biolabs). About 200 ng of individual libraries were pooled into a single tube and size selection (230~250 bp) of pooled libraries was performed using 100µL aliquot of pooled libraries onto a 2% agarose gel and MinElute Gel Extraction Kit (Qiagen).

Template preparation was done on the Ion OneTouch2 instrument using the Ion PGM Template OT2 200 Kit, followed by sequencing on an Ion Torrent PGM sequencer using the Ion PGM Sequencing 200 Kit v2 (Life Technologies), aiming for mean depth of 500X.

Simulation of NGS reads

In order to estimate the specificity of needlestack in multiple sequencing scenarios, we simulated NGS reads without any variant using NEAT-genreads (7). NEAT-genreads has the advantage to let the error rate vary across the positions following a model that was previously trained on real data. NEAT-genread was launched with default parameters except the read length that was set at 100, the insert size of paired reads at 250 with a variance of 10, and the proportion of variant reads at 0. In order to account for multiple sequencing scenarios, we generated four independent sets of NGS reads with different average coverages. For each scenario, 50 samples were generated from the genomic positions of the 20 most mutated genes in lung cancers (*AKT1*, *ALK*, *BRAF*, *CDKN2A*, *CTNNB1*, *EGFR*, *ERBB2*, *HRAS*, *KIT*, *KRAS*, *MET* ,*MTOR*, *NRAS*, *PDGFRA*, *PIK3CA*, *PTEN*, *PTGS2*, *RB1*, *STK11*, *TP53*).

Bioinformatics processing

Short reads from NGS sequencing were aligned to the hg19 human reference genome using the Torrent Suite software (v4.4.2) with default parameters. Somatic mutations were detected with needlestack using the version 1.0 and a QVAL threshold at 50. As recommended by Martincorena *et al.* (8), we used a threshold of 20 for the shearwaterML statistic.

Only cfDNA mutations detected in a high confidence base change were considered. A high confidence base change satisfied $P<0.05$ with P the probability that the number of duplicated mutations under the null hypothesis would be greater than or equal to the observed number of duplicated mutations, and is given by:

$$\mathrm{P}=\sum_{\mathrm{p}=\mathrm{p_{obs}}}^{p_{\max}} C_p$$

with C_p corresponding to the probability of observing p pairs when randomly picking k elements from a total of $2N$ paired elements calculated as:

$$C_p = \prod_{\mathrm{i}=0}^{p-1} \binom{k-2i}{2} \left[\prod_{\mathrm{j}=0}^{k-p-1} (2N-2j) \right] \frac{1}{\mathrm{p!k!}\binom{2N}{k}}$$

with *N* the number of sequenced samples in duplicates (2N sequenced libraries in total), *k* the number of libraries with a mutation called by needlestack, p_{obs} the number of duplicated called mutations, and p_{max} the total number of possible pairs when picking up *k* elements from a total of *N* pairs of elements. The detailed source code for cfDNA mutation analysis including all quality filtering step description is available on GitHub at: https://github.com/IARCbioinfo/target-seq.

For the germline analysis, GATK-HC variant calling was performed using version 3.4 and the HaplotypeCaller algorithm, followed by the joint genotyping step (9). Finally, Variant Quality Score Recalibration following the GATK best practices was applied, using dbSNP 138, HapMap 3.3, 1000 Genomes phase 1 and OMNI 2.5 databases. Options provided were "-tranche 100 -tranche 99.9 -tranche 99.0 -tranche 90.0" for both INDEL and SNP modes. GATK-HC variant calls were filtered on PASS and on Phred-scaled likelihood (9) larger than 20 and on VAF>0.1. BAM files were locally reassembled with ABRA version 1.0 (10) before launching the variant calling by needlestack. Needlestack germline calling was launched using our default germline parameters, *i.e.* QVAL>20, VAF>0.1 and the option *–-extra-robust*. This option helps needlestack to correctly estimate the error rate by excluding common germline variants (defined when more than 10% of the samples with a VAF higher than 20%) that tend to bias this estimation towards high values. For each of these base changes independently, this process first eliminates these germline samples and then estimates the error rate on remaining samples. In this germline analysis, both positions and variants with respectively a median coverage and an individual coverage less than 50 were removed from the whole analysis. Coverages were computed with samtools mpileup, counting only reads with a mapping quality higher than 20 and a base quality higher than 13. We considered as variant frequency the maximum proportion of samples carrying the variant estimated by both methods and then filtered out germline variants with a frequency higher than 10% to consider only rare variations.

Computation of ShearwaterML statistic used in the BAMsurgeon *in-silico* simulations

We launched the shearwaterML algorithm on the simulation data sets to compare its global performance with needlestack. As recommended by Martincorena *et al.* (8), we used a threshold of 20 for the shearwaterML statistic. We used default thresholds except that we increased *maxvaf* to 1 to call all mutations, and set *truncate* to 0.005 to avoid true mutations present initially at low VAF to enter the background error model and potentially reduce the sensitivity, as recommended in Martinorena *et al.* (8). ShearwaterML produced *p*-values instead of the shearwater Bayes factor, that we corrected for multiple testing using the Benjamini-Hochberg method which produces then *Q*-values that we finally transformed into Phred scale *Q*-values (QVAL).

Computation of a machine-learning model to predict the sensitivity of needlestack

In order to predict the sensitivity of needlestack in multiple sequencing scenarios, we have trained a random forest algorithm on mutations generated with BAMsurgeon (~ 10,000 mutations) using the R randomForest package (11). The model was trained with two key features: the number of mutated reads (AO=DP*VAF) and the ratio between the VAF and the sequencing error rate. To estimate the accuracy of our trained model, we have perform a *k*-fold cross validation with *k*=10, *i.e.* we have split

our mutation data into 10 subsets and for each subset we have trained our model on the rest of mutations and have applied it on the subset mutations. With this method, we obtained for each of the 10,000 mutations a predicted probability that needlestack can detected it. Finally, we compared this to the observed detection of needlestack in order to compute a precision-recall curve and its area under the curve corresponding to the accuracy of the predictive model. Once this model has been trained and the accuracy has been estimated, we have used it to compute the expected sensitivity of needlestack depending on these same two key parameters. Of note, we also followed the same procedure using a logistic regression classifier that resulted in a slightly decreased AUC than the Random Forest (0.992 *vs.* 0.998, respectively).

Using needlestack to compute the error rate distribution

Needlestack can be forced to compute the error rate for the three possible single nucleotide changes at every query position (i.e. to output the results of the error model even when no variants are identified). This can be achieved by launching needlestack with parameters --all_SNVs, --min_ao 0 and --min_dp 1. This way, in our analysis, the 5112 error rates across the *TP53* gene were computed.

REFERENCES

1. Aeberhard, W.H., Cantoni, E. and Heritier, S. (2014) Robust inference in the negative binomial regression model with an application to falls data. *Biometrics*, **70**, 920-931.
2. Di Tommaso, P., Chatzou, M., Floden, E.W., Barja, P.P., Palumbo, E. and Notredame, C. (2017) Nextflow enables reproducible computational workflows. *Nature biotechnology*, **35**, 316-319.
3. Perkel, J. (2016) Democratic databases: science on GitHub. *Nature*, **538**, 127-128.
4. Merkel, D. (2014) Docker: lightweight Linux containers for consistent development and deployment. *Linux J.*, **2014**, 2.
5. Gruning, B., Dale, R., Sjodin, A., Chapman, B.A., Rowe, J., Tomkins-Tinch, C.H., Valieris, R. and Koster, J. (2018) Bioconda: sustainable and comprehensive software distribution for the life sciences. *Nature methods*, **15**, 475-476.
6. Nong, J., Gong, Y., Guan, Y., Yi, X., Yi, Y., Chang, L., Yang, L., Lv, J., Guo, Z., Jia, H. *et al.* (2018) Circulating tumor DNA analysis depicts subclonal architecture and genomic evolution of small cell lung cancer. *Nat Commun*, **9**, 3114.
7. Stephens, Z.D., Hudson, M.E., Mainzer, L.S., Taschuk, M., Weber, M.R. and Iyer, R.K. (2016) Simulating Next-Generation Sequencing Datasets from Empirical Mutation and Sequencing Models. *PloS one*, **11**, e0167047.
8. Martincorena, I., Fowler, J.C., Wabik, A., Lawson, A.R.J., Abascal, F., Hall, M.W.J., Cagan, A., Murai, K., Mahbubani, K., Stratton, M.R. *et al.* (2018) Somatic mutant clones colonize the human esophagus with age. *Science (New York, N.Y.)*, **362**, 911-917.
9. Poplin, R., Ruano-Rubio, V., DePristo, M.A., Fennell, T.J., Carneiro, M.O., Van der Auwera, G.A., Kling, D.E., Gauthier, L.D., Levy-Moonshine, A., Roazen, D. *et al.* (2018) Scaling accurate genetic variant discovery to tens of thousands of samples. *bioRxiv*, 201178.
10. Mose, L.E., Wilkerson, M.D., Hayes, D.N., Perou, C.M. and Parker, J.S. (2014) ABRA: improved coding indel detection via assembly-based realignment. *Bioinformatics (Oxford, England)*, **30**, 2813-2815.
11. Liaw, A. and Wiener, M. (2002) Classification and Regression by RandomForest. *R News*, **2(3)**, 18-22, https://CRAN.R-project.org/doc/Rnews/.

SUPPLEMENTARY TABLE AND FIGURE LEGENDS

Supplementary Figure S1: needlestack workflow description. The first step corresponds to the creation of a BED file containing the DNA positions on which the calling should be launched using the fasta index of the input reference genome; this step is optional, only performed if target positions are not provided by the user. The second step splits the BED file into multiple sets of positions to run the algorithm independently on each set in parallel; the number of position sets is given as an input by the user. The third step runs the variant calling from three piped substeps: (i) the mpileup file building using samtools, (ii) the parsing of the mpileup to produce count data per sample in a tabulated readable file, (iii) and the robust regression in R on each tested mutation. The fourth and last step merges VCF files previously produced in parallel and outputs the global result. Workflow orchestration is done thanks to the nextflow domain specific language.

Supplementary Figure S2: estimated error rate distributions from amplicon-based sequencing of *TP53* gene (median coverage around 10,000X). Distributions of error rates are shown for each of the 96 possible base variation (with flanking 3' and 5' bases), and are colored by DNA base changes.

Supplementary Figure S3: estimated error rate distributions for both SNVs and indels from the same data as used in supplementary Figure 2. (A) Error rate distributions are shown as a function of the type of SNV (yellow for transversions and green for transitions), the length of the insertion (pink) and the length of the deletion (blue), with *n* indicating the total number of error rates used to compute the distribution. (B) Error rate distributions restricted to insertions and deletions, as a function of the size of the homopolymer region (i.e. depending on the number of repeated nucleotides) at the position.

Supplementary Figure S4: (A) Precision-recall curve based on a *k*-fold cross validation (with *k*=10) of a random forest algorithm predicting the sensitivity of needlestack for multiple types of sequencing scenarios. The area under the precision-recall curve (AUC) was estimated at 0.998. (B) Prediction of the sensitivity of needlestack from the trained random forest algorithm.

Supplementary Figure S5: Number of false positives per megabase (y axis) given the QVAL threshold (x axis) of needlestack, based on the simulation of NGS reads for four distinct sequencing scenarios with variable coverage (DP).

Supplementary Figure S6: validation of cfDNA mutations in the matched tumour sample for a total of 11 SCLC cases and 24 SCC cases. (A) Correlation of cfDNA and tumour VAF for deleterious validated mutations (total=12). Pearson correlation coefficient ρ was estimated as 0.59. Grey dashed line corresponds to the fitted linear regression characterized by the values *a* (slope) and *b* (intercept). (B) Needlestack regression plots of a validated deleterious SNV (red dot in A). Left panel corresponds to cfDNA data and right panel to tumour data.

Supplementary Figure S7: (A) Distribution of the sequencing error rates for SNVs detected by GATK-HC but not detected by needlestack from 62 WES samples (total of 1385 mutations), estimated using kernel density estimation. (B) Needlestack regression plots for one particular position where GATK called 3 variants (in purple). Needlestack estimated a high sequencing error rate (around 1%) for this mutation and therefore did not call it, highlighting the fact that estimating the systematic error rate across multiple sample should reduce the false discovery rate of the method.

Supplementary Figure S8: sensitivity of needlestack as a function of the VAF for the *in-silico* simulated insertions (A) and deletions (B), depending on the error rate for the alteration. Cumulative number of false discoveries per sample is shown for insertions (C) and deletions (D) per sample, depending on the VAF of the detected mutation. This number was computed firstly for all detected indels (the result is per library) and secondly for indels validated in the second library (the result is per sample). Dashed lines correspond to the number of indels not introduced by BAMsurgeon that are however not in strand bias (RVSB<0.85).

Supplementary Table S1: description of the 22 mutations identified by needlestack in the cfDNA of 35 lung cancer patients.

Supplementary Table S2: variant status and genotype attributed by needlestack as a function of variant detection and the power to detect variants in tumour and matched normal samples.

Supplementary Figure 1.

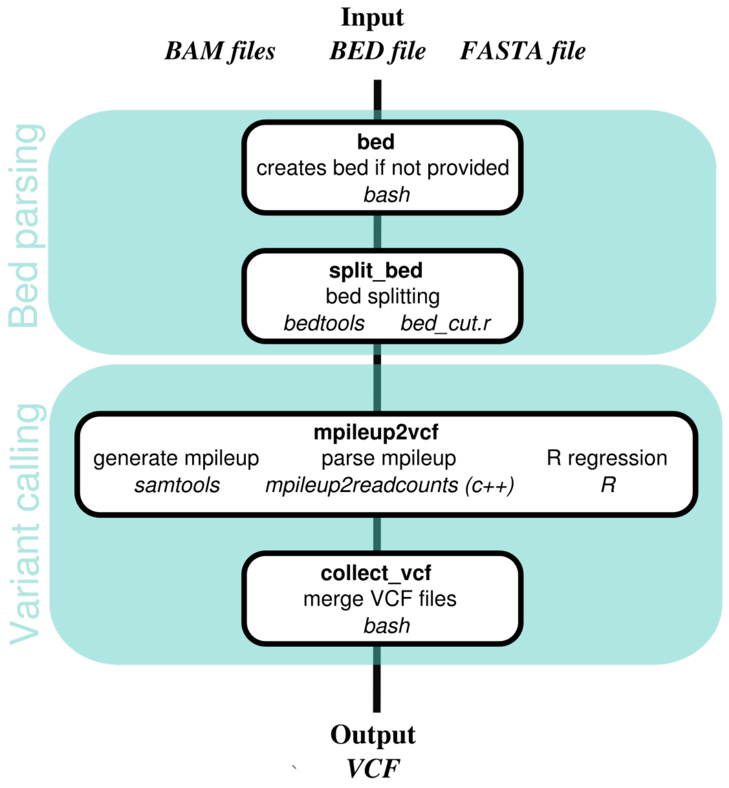

Supplementary Figure 2.

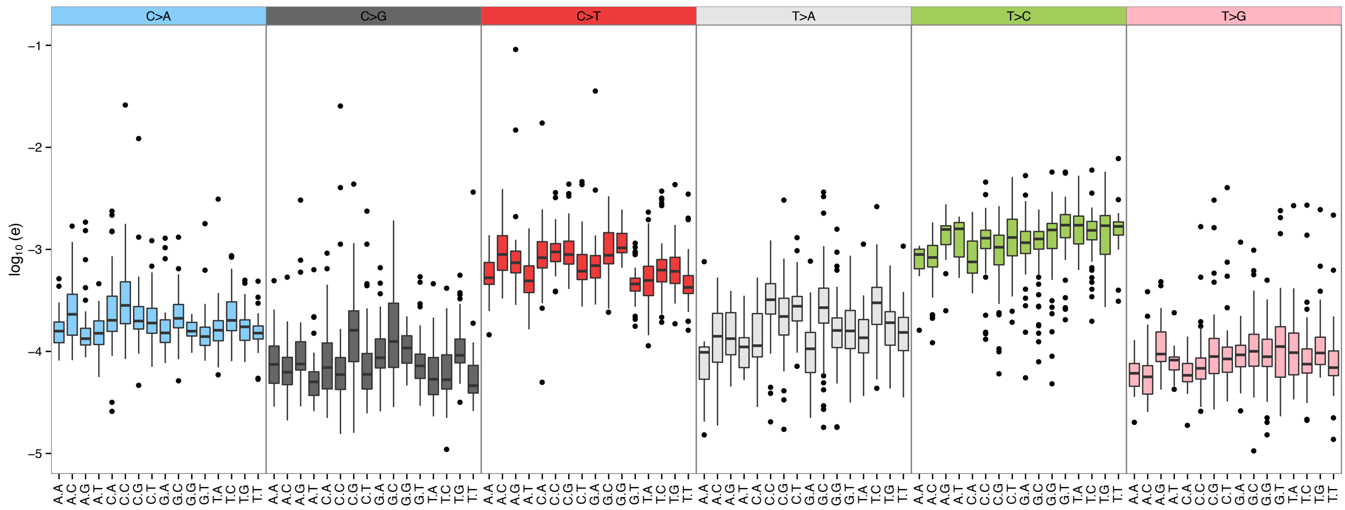

Supplementary Figure 3.

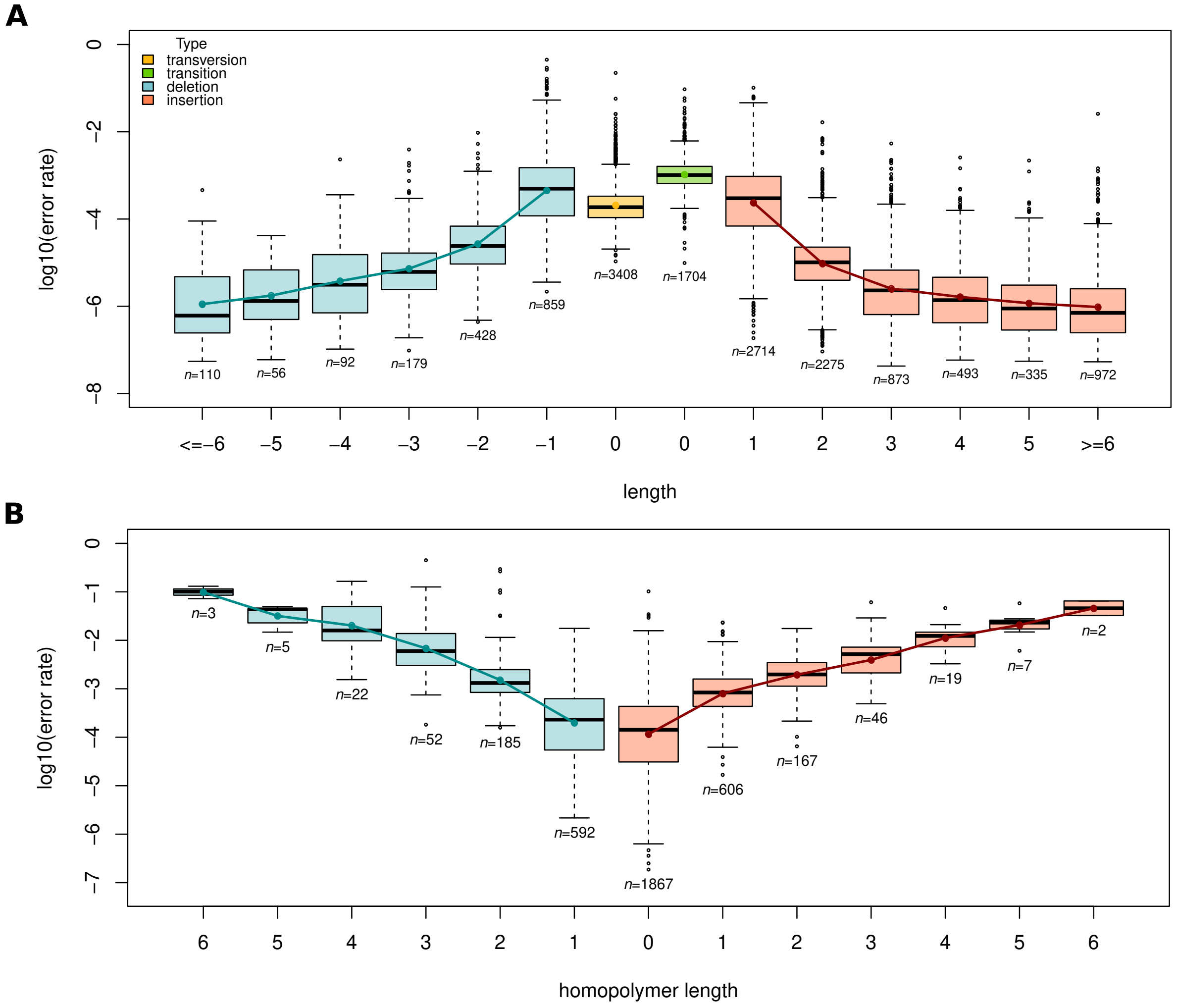

Supplementary Figure 4.

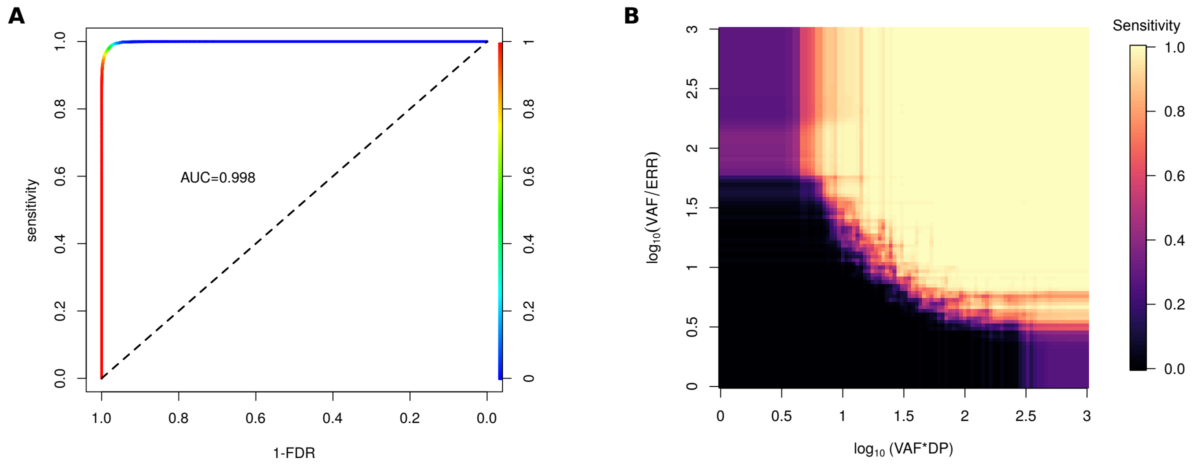

Supplementary Figure 5.

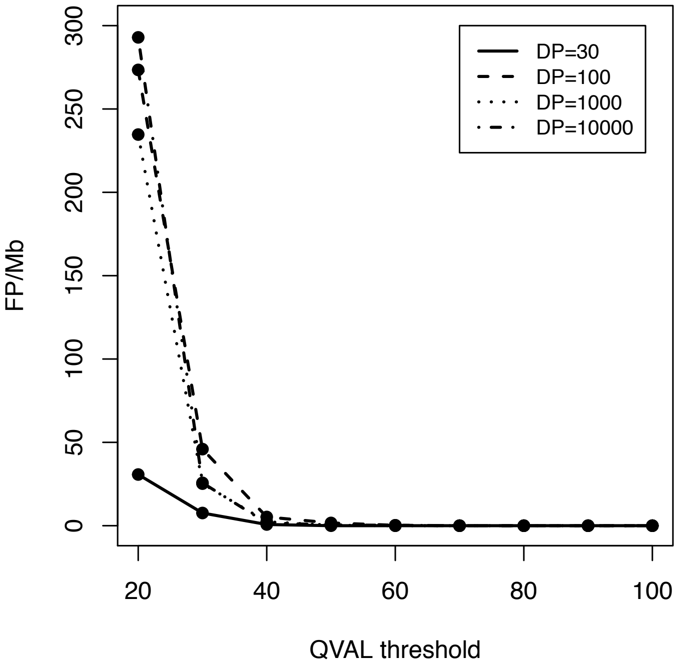

Supplementary Figure 6.

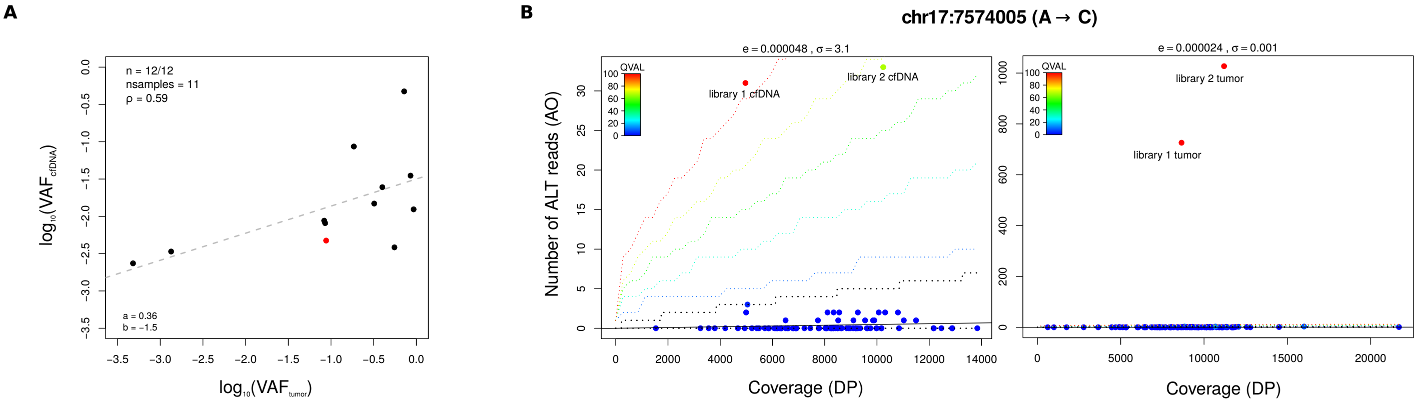

Supplementary Figure 7.

A

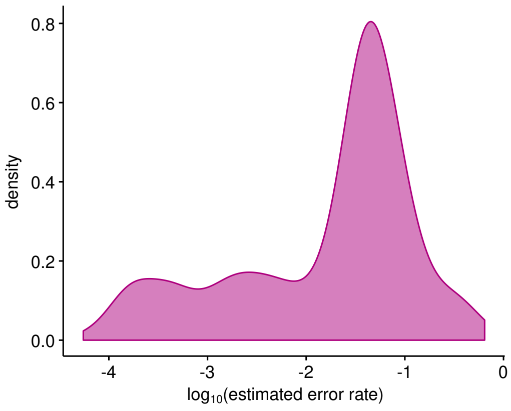

B

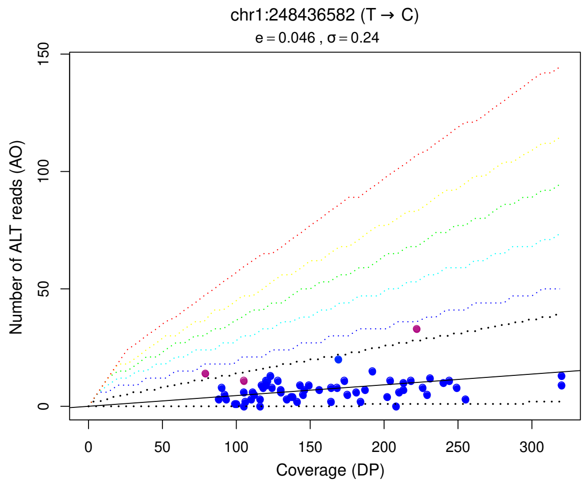

Supplementary Figure 8.

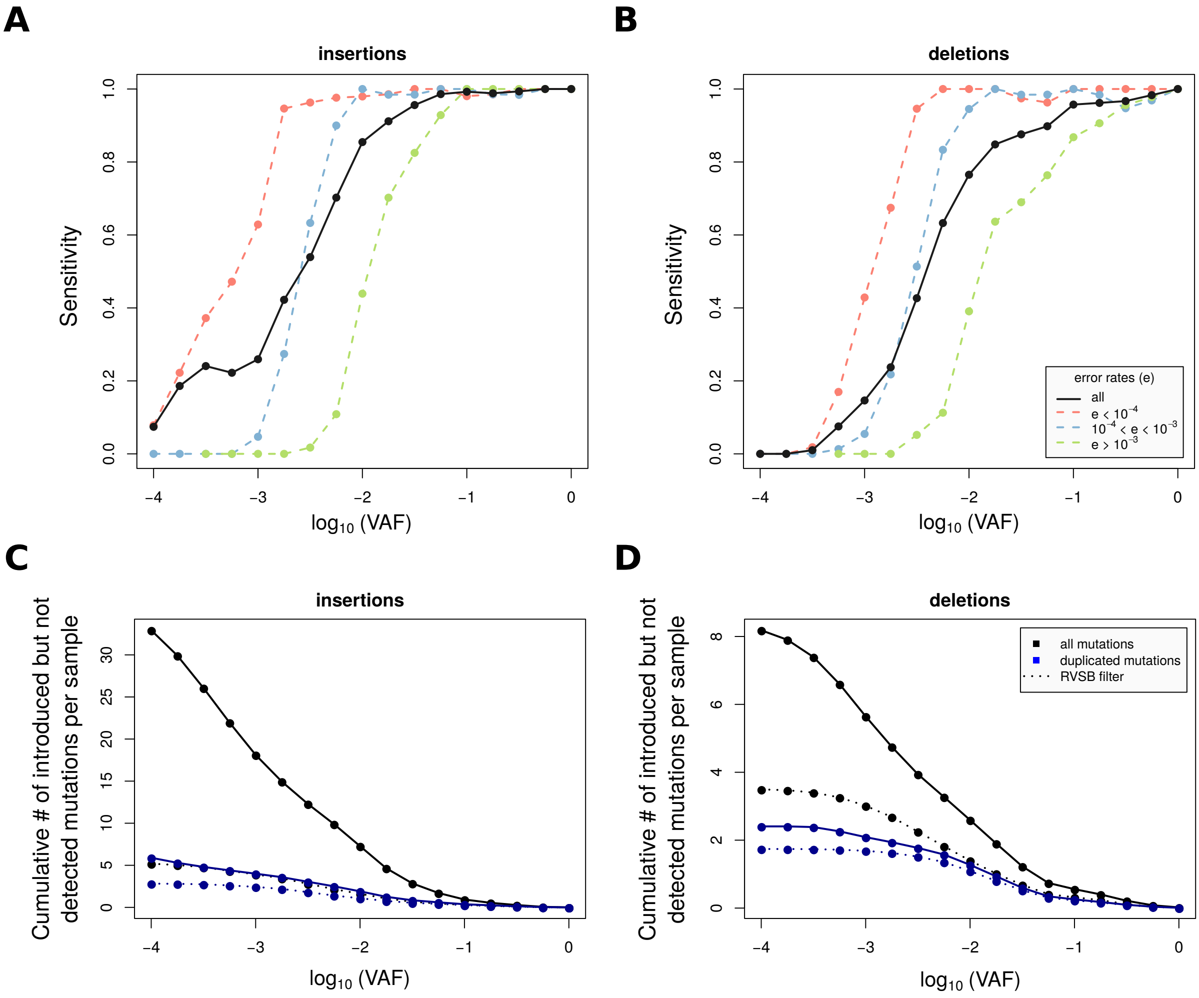

Supplementary Table 1.

See attached Excel file

Supplementary Table 2.

**Variant in Normal**	**Power to detect variant in Normal**	**Variant in Tumor**	**Power to detect variant in Tumor**	**STATUS**	**Normal GT**	**Tumor GT**
no	no	no	no	.	./.	./. *
no	no	no	**YES**	.	./.	0/0 *
no	no	**YES**	**YES** or no	UNKNOWN	./.	0/1 or 1/1
no	**YES**	no	no	.	0/0	./. *
no	**YES**	no	**YES**	.	0/0	0/0 *
no	**YES**	**YES**	**YES** or no	SOMATIC †	0/0	0/1 or 1/1
**YES**	**YES** or no	no	no	GERMLINE_UNCONFIRMABLE	0/1 or 1/1	./.
**YES**	**YES** or no	no	**YES**	GERMLINE_UNCONFIRMED	0/1 or 1/1	0/0
**YES**	**YES** or no	**YES**	**YES** or no	GERMLINE_CONFIRMED	0/1 or 1/1	0/1 or 1/1

* power is computed using a binomial distribution with mean $power\_min\_af$ (default value is 0.01)

† only in Tumor; Normal STATUS is “."
